# Risk factors analysis of surgical site infections in postoperative colorectal cancer: a nine-year retrospective study

**DOI:** 10.1186/s12893-023-02231-z

**Published:** 2023-10-24

**Authors:** Cong Han, Wei Chen, Xiao-Li Ye, Fei Cheng, Xin-You Wang, Ai-Bin Liu, Zai-Hu Mu, Xiao-Jun Jin, Yan-Hong Weng

**Affiliations:** Department of Surgery, Huangshan Shoukang Hospital, 58 Meiling Rd, Huangshan, 245000 China

**Keywords:** Surgical site infections, Colorectal cancer, Risk factors, Incidence

## Abstract

**Background:**

Colorectal cancer (CRC) patients undergoing surgery are at a high risk of developing surgical site infections (SSIs), which contribute to increased morbidity, prolonged hospitalization, and escalated healthcare costs. Understanding the incidence, risk factors, and impact of SSIs is crucial for effective preventive strategies and improved patient outcomes.

**Methods:**

This retrospective study analyzed data from 431 CRC patients who underwent surgery at Huangshan Shoukang Hospital between 2014 and 2022. The clinical characteristics and demographic information were collected. The incidence and impact of SSIs were evaluated, and independent risk factors associated with SSIs were identified using multivariable logistic regresison. A nomogram plot was constructed to predict the likelihood of SSIs occurrence.

**Results:**

The overall incidence rate of SSIs was 7.65% (33/431). Patients with SSIs had significantly longer hospital stays and higher healthcare costs. Risk factors for SSIs included elevated Body Mass Index (BMI) levels (odds ratio, 1.12; 95% CI, 1.02—1.23; *P* = 0.017), the presence of diabetes (odds ratio, 3.88; 95% CI, 1.42 – 9.48; *P* = 0.01), as well as specific surgical factors such as open surgical procedures (odds ratio, 2.39; 95% CI [1.09; 5.02]; *P* = 0.031), longer surgical duration (odds ratio, 1.36; 95% CI [1.01; 1.84]; *P* = 0.046), and the presence of a colostomy/ileostomy (odds ratio, 3.17; 95% CI [1.53; 6.62]; *P* = 0.002). Utilizing multivariable regression analysis, which encompassed factors such as open surgical procedures, the presence of diabetes and colostomy/ileostom, the nomogram plot functions as a visual aid in estimating the individual risk of SSIs for patients.

**Conclusions:**

Risk factors for SSIs included higher BMI levels, the presence of diabetes, open surgical procedures, longer surgical duration, and the presence of colostomy/ileostomy. The nomogram plot serves as a valuable tool for risk assessment and clinical decision-making.

## Background

Colorectal cancer (CRC) is a prevalent malignancy globally, with an incidence rate ranging from 16.1 to 45.3 per 100,000 and an mortality rate ranging from 9.0 to 16.1 per 100000 [1, 2]. According to the latest 2023 estimates from the American Cancer Society, there will be approximately 153,020 new cases and 52,550 deaths from CRC, making it the third most common cancer diagnosis and second leading cause of cancer mortality [2]. Surgical intervention is the primary therapeutic approach for CRC patients. However, the presence of a large microbial population in the rectum and colon, coupled with the potential for bacterial growth facilitated by surgery, renders these patients particularly susceptible to developing surgical site infections (SSIs) [3, 4]. Studies have reported an SSIs incidence ranging from 1.5% to 8.8% following colorectal surgery [4–6]. Despite advancements in surgical techniques and perioperative care, the management of SSIs remains a significant challenge in CRC patients [7, 8]. SSIs contribute to increased morbidity, prolonged hospitalization, and escalated healthcare costs [9–11]. Therefore, understanding the incidence, risk factors, and impact of SSIs is crucial for developing effective preventive strategies and improving patient outcomes [12, 13].

In fact, the majority of SSIs can be prevented through preventive measures [14, 15]. Numerous related factors have been reported, with some considered strong predictors of SSIs occurrence, such as high body mass index (BMI) and diabetes [16–18]. However, it has been reported that during the COVID-19 pandemic, there has been a decrease in SSIs in neurosurgical procedures [19]. This effect needs further investigation in CRC patients. Currently, research on the incidence of SSIs and related risk factors in CRC patients is relatively limited, especially in China [20–22]. Therefore, this retrospective study aimed to investigate the occurrence of SSIs in postoperative CRC patients and analyze the associated risk factors.

In this study, we analyzed data from a 9-year period of CRC surgery patients at our institution, aiming to determine the incidence of SSIs following colorectal surgery and identify associated risk factors. Additionally, we developed a nomogram figure as a practical tool for clinical decision-making. Our research findings will contribute to optimizing the management of CRC surgical patients and mitigating the burden of SSIs.

## Methods

### Study design and participants

This retrospective study included patients who underwent CRC surgery between 2014 and 2022 at Department of Surgery, Huangshan Shoukang Hospital. A total of 431 patients were included in the analysis. The inclusion criteria for this study were as follows: (1) age over 18 years, (2) confirmed histological diagnosis of CRC scheduled for elective colorectal resection, (3) absence of incisions other than the abdomen or perineum, and (4) no artificial implants. The study population included patients undergoing extended resections, such as pelvic exenteration, and combined resections of other intra-abdominal organs such as the stomach, liver, and pancreas. The protocol for this study was approved by the Institutional Review Board of Huangshan Shoukang Hospital.

### Data collection

Data collection was performed by trained researchers using standardized data collection forms. Data collection was performed by trained researchers using standardized data collection forms. Demographic variables included gender, age, height, weight, and BMI. Clinical variables were COVID-19 occurrence, hypertension, diabetes, hepatitis B, gallstones, surgical history, tumor location, tumor stage, preoperative chemotherapy, surgical start time (days), hair removal, bowel preparation and methods (none, oral antibiotics only (ORAB), mechanical bowel preparation only (MBP), or combined mechanical and oral antibiotics (ORAB + MBP)), surgical wound class, surgeon seniority, surgical approach, surgical duration (hours), colostomy/ileostomy creation, and postoperative bowel obstruction. Our staging was performed using the American Joint Committee on Cancer Colorectal Cancer TNM Staging System (8th edition 2017) [23].

### Outcome measures

The primary outcome measure was the occurrence of SSIs. SSIs were categorized as superficial incisional, deep incisional, or organ/space [13, 24]. The incidence of SSIs was calculated as the number of cases divided by the total number of patients included in the study.

### Multivariable risk analysis and nomogram method

To identify the independent risk factors associated with SSIs occurrence, multivariable logistic regression analysis was conducted, included factors that exhibited significant differences in the univariate analysis. The odds ratios and their corresponding 95% confidence intervals (CIs) were reported.

The nomogram was developed using the "rms" package in R software (version 4.3.1) [25, 26]. The variables included in the nomogram were selected based on multivariable logistic regression analysis. The points assigned to each variable were determined by their regression coefficients, which were obtained from the multivariable logistic regression analysis. To use the nomogram, each variable's value is located on the corresponding axis, and a straight line is drawn upwards to determine the corresponding points for that variable. The points for all variables are summed, and a straight line is drawn downwards to the "Probability of SSIs" axis to estimate the individual probability of SSIs occurrence. The nomogram provides a user-friendly tool for clinicians to calculate the risk of SSIs in individual patients and make informed decisions regarding preventive interventions. It can also be used as a prognostic tool to assess the impact of different risk factors on SSIs occurrence.

### Statistical analysis

We employed R software (version 4.3.1) along with packages including autoReg, compareGroups, rms, and VRPM for data analysis. All results were summarized using descriptive statistics. Categorical variables were presented as percentages while continuous variables were reported as median and interquartile range (IQR). For normally distributed continuous data, differences between groups were assessed using independent sample t-tests. For non-normally distributed continuous data, the Mann–Whitney U test was utilized. Pearson's chi-square tests or Fisher's exact tests were applied for categorical variables. Multivariate logistic regression analysis was conducted to identify independent risk factors for SSIs. Variables found to be statistically significant (*P* < 0.05) in univariate analyses were included in the multivariate model. The threshold for statistical significance in all analyses was defined a priori as *P* < 0.05.

## Results

### Incidence and impact of SSIs in CRC patients

**Fig. 1:**
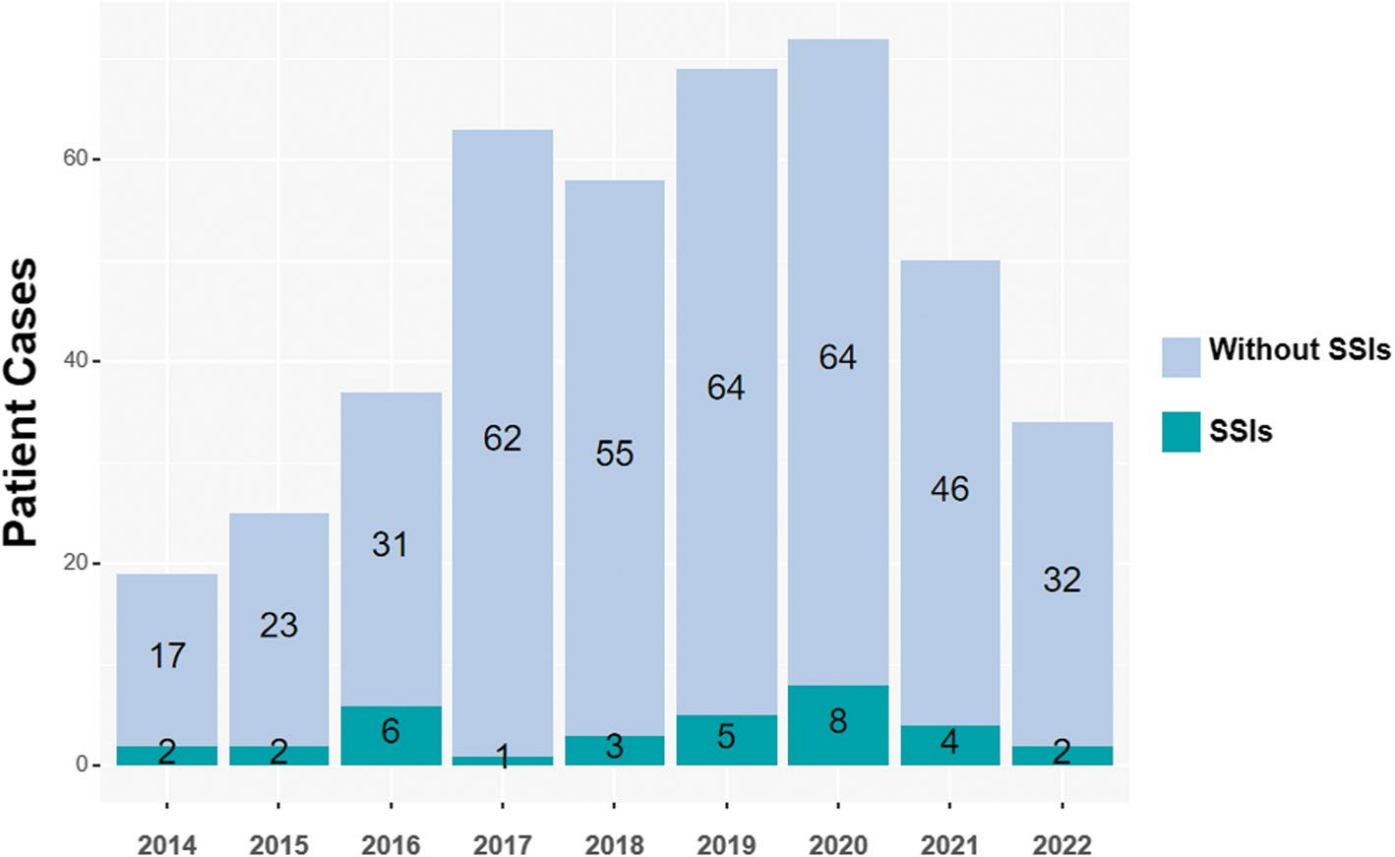
Incidence of SSIs in CRC Patients. This bar chart depicts the number of colorectal cancer surgery cases and the number of patients with SSIs annually from 2014 to 2022 over 9 years. "Without" refers to patients without occurrence of SSIs. "SSIs" represents colorectal cancer patients with postoperative SSIs

In this study, we investigated the occurrence of SSIs in postoperative CRC patients. A total of 431 patients who underwent CRC surgery between 2014 and 2022 were included in the analysis (Fig. 1). Among these patients, 33 cases were identified as SSIs, resulting in an overall incidence rate of 7.65%. The distribution of SSIs based on infection types revealed that 17 cases (17/33, 51.5%) were attributed to organ or organ space infections, while 12 cases (12/33, 36.4%) were associated with deep incisional, and 4 cases (3/33, 12.1%) were related to superficial incisional (Table 1).

**Table 1:** Types of postoperative SSIs in CRC patients

Types	SSIs (*N* = 33)
Superficial incisional SSIs	4 (12.1%)
Deep incisional SSIs	12 (36.4%)
Organ or organ space SSIs	17 (51.5%)

Patients who experienced SSIs had significantly longer hospital stays compared to those without SSIs (median: 24 days vs. 16 days, *p* < 0.001) (Table 2). Additionally, the presence of SSIs was associated with a significant increase in healthcare costs, with an average expenditure of 43,909 yuan compared to 32,635 yuan for patients without SSIs (*p* < 0.001) (Table 2). Our findings highlight the considerable occurrence of SSIs in postoperative CRC patients and underscore their detrimental effects on prolonged hospitalization and increased healthcare expenses.

**Table 2:** Impact of postoperative SSIs on hospitalization in CRC patients

	[ALL]	Without SSIs	SSIs	OR	p.OR	p.overall
	*N* = 431	*N* = 398 (92.4%)	*N* = 33 (7.6%)			
Length of hospital stay (Days)	17.0 [14.0;21.8]	16.0 [13.0;21.0]	24.0 [19.0;38.0]	1.07 [1.04;1.10]	< 0.001	< 0.001
Total cost (Chinese Yuan)	32,974 [28284;40445]	32,635 [28141;39413]	43,909 [34124;53769]	1.00 [1.00;1.00]	< 0.001	< 0.001

### The risk factors associated with postoperative SSIs in CRC patients

We conducted a comprehensive analysis of various demographic and clinical variables to identify factors associated with the occurrence of SSIs. The results are summarized in Table 3. Our analysis revealed several significant factors that were associated with an increased risk of postoperative SSIs. These factors included included higher BMI levels (odds ratio, 1.12; 95% CI, 1.02—1.23; *P* = 0.017), the presence of diabetes (odds ratio, 3.88; 95% CI, 1.42 – 9.48; *P* = 0.01), open surgical procedures (odds ratio, 2.39; 95% CI [1.09; 5.02]; *P* = 0.031), longer surgical duration (odds ratio, 1.36; 95% CI [1.01; 1.84]; *P* = 0.046), and the presence of a colostomy/ileostomy (odds ratio, 3.17; 95% CI [1.53; 6.62]; *P* = 0.002). These findings underscore the importance of considering these risk factors in the management and prevention of SSIs in CRC patients.

**Table 3:** Analysis of risk factors associated with postoperative SSIs in CRC patients

	**[ALL] (*N* = 431)**	**Without SSIs (*N* = 398)**	**SSIs (*N* = 33)**	**OR**	**p.OR**	**p.Overall**
**Gender**						0.476
Male	269 (62.4%)	246 (61.8%)	23 (69.7%)	Ref	Ref	
Female	162 (37.6%)	152 (38.2%)	10 (30.3%)	0.71 [0.31;1.50]	0.379	
**Age**	66.0 [56.0;72.0]	66.0 [57.0;72.0]	65.0 [55.0;71.0]	0.99 [0.96;1.02]	0.48	0.409
**Height**	162 [155;168]	162 [155;168]	160 [156;169]	1.01 [0.96;1.05]	0.77	0.948
**Weight**	57.0 [50.0;65.0]	57.0 [50.0;65.0]	62.3 [54.0;68.0]	1.03 [1.00;1.07]	0.041	0.053
**BMI**	21.9 [19.9;24.0]	21.8 [19.8;24.0]	23.3 [21.5;24.6]	1.12 [1.02;1.23]	**0.017**	**0.026**
**COVID-19 pandemic**						0.578
No	274 (63.6%)	255 (64.1%)	19 (57.6%)	Ref	Ref	
Yes	157 (36.4%)	143 (35.9%)	14 (42.4%)	1.32 [0.63;2.71]	0.461	
**Hypertension**						0.932
No	291 (67.5%)	268 (67.3%)	23 (69.7%)	Ref	Ref	
Yes	140 (32.5%)	130 (32.7%)	10 (30.3%)	0.90 [0.40;1.92]	0.798	
**Diabetes**						**0.008**
No	398 (92.3%)	372 (93.5%)	26 (78.8%)	Ref	Ref	
Yes	33 (7.66%)	26 (6.53%)	7 (21.2%)	3.88 [1.42;9.48]	0.01	
**HepatitisB**						1
No	388 (90.0%)	358 (89.9%)	30 (90.9%)	Ref	Ref	
Yes	43 (9.98%)	40 (10.1%)	3 (9.09%)	0.93 [0.21;2.80]	0.914	
**Gallstones**						1
No	400 (92.8%)	369 (92.7%)	31 (93.9%)	Ref	Ref	
Yes	31 (7.19%)	29 (7.29%)	2 (6.06%)	0.88 [0.13;3.13]	0.861	
**Surgical history**						0.502
No	396 (91.9%)	364 (91.5%)	32 (97.0%)	Ref	Ref	
Yes	35 (8.12%)	34 (8.54%)	1 (3.03%)	0.38 [0.02;1.85]	0.282	
**Tumor location**						0.184
Colon	237 (55.0%)	223 (56.0%)	14 (42.4%)	Ref	Ref	
Rectum	194 (45.0%)	175 (44.0%)	19 (57.6%)	1.72 [0.84;3.62]	0.138	
**Tumor stage**						0.884
I	68 (15.8%)	64 (16.1%)	4 (12.1%)	Ref	Ref	
II	127 (29.5%)	118 (29.6%)	9 (27.3%)	1.20 [0.37;4.71]	0.776	
III	190 (44.1%)	173 (43.5%)	17 (51.5%)	1.53 [0.54;5.62]	0.451	
IV	46 (10.7%)	43 (10.8%)	3 (9.09%)	1.13 [0.20;5.65]	0.883	
**Preoperative chemotherapy**						0.659
No	411 (95.4%)	380 (95.5%)	31 (93.9%)	Ref	Ref	
Yes	20 (4.64%)	18 (4.52%)	2 (6.06%)	1.45 [0.20;5.40]	0.654	
**Surgical start time (days)**	5.00 [3.00;7.00]	5.00 [3.00;7.00]	5.00 [4.00;8.50]	1.09 [1.01;1.18]	0.024	0.281
**Hair removal**						1
No	354 (83.1%)	327 (83.0%)	27 (84.4%)	Ref	Ref	
Yes	72 (16.9%)	67 (17.0%)	5 (15.6%)	0.93 [0.30;2.32]	0.879	
**Bowel preparation**						0.589
No	97 (22.7%)	88 (22.3%)	9 (28.1%)	Ref	Ref	
Yes	330 (77.3%)	307 (77.7%)	23 (71.9%)	0.73 [0.33;1.72]	0.45	
**Bowel preparation methods**						0.833
None	16 (3.71%)	15 (3.77%)	1 (3.03%)	Ref	Ref	
OABP only	79 (18.3%)	72 (18.1%)	7 (21.2%)	1.31 [0.20;35.2]	0.811	
MBP only	9 (2.09%)	8 (2.01%)	1 (3.03%)	1.83 [0.04;77.6]	0.72	
MBP + OABP	327 (75.9%)	303 (76.1%)	24 (72.7%)	1.05 [0.20;26.2]	0.962	
**Surgical wound class**						0.169
Clean-contaminated	390 (92.0%)	362 (92.6%)	28 (84.8%)	Ref	Ref	
Contaminated or dirty	34 (8.02%)	29 (7.42%)	5 (15.2%)	2.27 [0.72;5.95]	0.151	
**Grade of lead surgeon**						0.074
Senior	315 (73.1%)	286 (71.9%)	29 (87.9%)	Ref	Ref	
Middle	116 (26.9%)	112 (28.1%)	4 (12.1%)	0.36 [0.10;0.96]	0.04	
**Surgical approach**						**0.036**
Laparoscopic	342 (79.4%)	321 (80.7%)	21 (63.6%)	Ref	Ref	
Open	89 (20.6%)	77 (19.3%)	12 (36.4%)	2.39 [1.09;5.02]	0.031	
**Surgical duration (hours)**	3.17 [2.45;3.92]	3.11 [2.42;3.83]	3.63 [2.83;4.20]	1.36 [1.01;1.84]	0.046	**0.021**
**Colostomy/ileostomy**						**0.002**
No	304 (70.5%)	289 (72.6%)	15 (45.5%)	Ref	Ref	
Yes	127 (29.5%)	109 (27.4%)	18 (54.5%)	3.17 [1.53;6.62]	0.002	
**Postoperative bowel obstruction**						1
No	399 (92.6%)	368 (92.5%)	31 (93.9%)	Ref	Ref	
Yes	32 (7.42%)	30 (7.54%)	2 (6.06%)	0.84 [0.12;3.01]	0.822	

### Impact of surgical approach and diabetes on the occurrence of SSIs

Subsequently, we conducted separate analyses to examine the impact of surgical approach and the presence of diabetes on the occurrence and types of SSIs (Tables 4 and 5). Our findings revealed that open surgical procedures and the presence of diabetes significantly increased the incidence of incision or ostomy site infections (Tables 4 and 5). Taking into account the controversial impact of bowel preparation and its methods on SSIs, we compared the incidence of SSIs among four groups: None, OAMP, MBP, and OAMP + MBP. Our findings revealed no significant differences among these groups (Table 6).

**Table 4:** Impact of surgical approach on the occurrence of SSIs

Surgical Approach	[ALL]	Laparoscopic	Open	P
	*N* = 431	*N* = 342	*N* = 89	
**SSIs**	33 (7.66%)	21 (6.14%)	12 (13.5%)	0.036
**SSIs categories**				0.032
Superficial incisional	402 (93.3%)	324 (94.7%)	78 (87.6%)	
Deep incisional	29 (6.73%)	18 (5.26%)	11 (12.4%)	
Organ or organ space	3 (0.70%)	2 (0.58%)	1 (1.12%)

**Table 5:** Impact of diabetes on the occurrence of SSIs

Diabetes	[ALL]	With	Without	*P*
	*N* = 431	*N* = 398	*N* = 33	
**SSIs**	33 (7.66%)	26 (6.53%)	7 (21.2%)	0.008
**SSIs categories**				0.495
Superficial incisional	4 (12.1%)	4 (15.4%)	0 (0.00%)	
Deep incisional	12 (36.4%)	8 (30.8%)	4 (57.1%)	
Organ or organ space	17 (51.5%)	14 (53.8%)	3 (42.9%)

**Table 6:** Impact of bowel preparation methods on the occurrence of SSIs

	**[ALL]**	**None**	**OABP only**	**MBP only**	**MBP + OABP**	**P**
	*N* = 431	*N* = 16	*N* = 79	*N* = 9	*N* = 327	
**SSIs (YES)**	33 (7.66%)	1 (6.25%)	7 (8.86%)	1 (11.1%)	24 (7.34%)	0.833
**SSIs categories**						0.396
Superficial incisional	4 (12.1%)	0 (0.00%)	2 (28.6%)	0 (0.00%)	2 (8.33%)	
Deep incisional	12 (36.4%)	0 (0.00%)	1 (14.3%)	0 (0.00%)	11 (45.8%)	
Organ or organ space	17 (51.5%)	1 (100%)	4 (57.1%)	1 (100%)	11 (45.8%)	

### The multivariable risk and nomogram analysis

Subsequently, to identify the independent risk factors for SSIs following colorectal surgery, we conducted a multivariable logistic regression analysis. The results revealed that having diabetes, undergoing an open surgical approach, and having a colostomy/ileostomy remained significant independent risk factors associated with SSIs occurrence (see Table 7). Higher BMI levels and longer surgical duration are not independent risk factors.

**Table 7:** Multivariate analysis

	Univariable	P.Univariable	Multivariable	P.Multivariable
**Body Mass Index**	1.12 [1.02;1.23]	0.017	1.11 [0.99;1.25]	0.069
**Diabetes**				**0.008**
No	Ref	Ref		
Yes	3.88 [1.42;9.48]	0.01	3.78 [1.41;10.12]	
**Surgical approach**				**0.039**
Laparoscopic	Ref	Ref		
Open	2.39 [1.09;5.02]	0.031	2.45 [1.05;5.75]	
**Colostomy/ileostomy**				**0.007**
No	Ref	Ref		
Yes	3.17 [1.53;6.62]	0.002	3.01 [1.34;6.75]	
**Surgical duration**	1.36 [1.01;1.84]	0.046	1.17 [0.83;1.64]	0.362

**Fig. 2:**
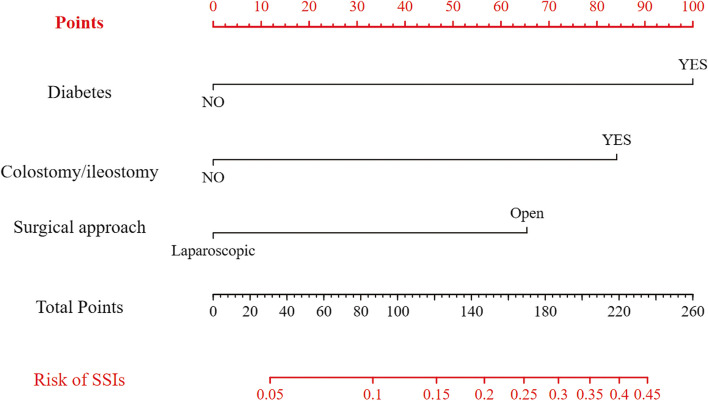
Nomogram for predicting the probability of SSIs after CRC surgery. Components of the nomogram:Variables (Diabetes, Colostomy/ileostomy, Surgical approach); Points, including Points for each variable and Total Points in the plot; Predicted Probability based on the Total Points; By drawing a vertical line on the scale of each variable, a score can be assigned to them, and this total score can predict the probability of SSIs occurrence

Based on the results of multivariable logistic regression analysis, we constructed a Nomogram plot (Fig. 2). The Nomogram plot is a visual tool used to estimate the individual risk of developing SSIs in patients. It is based on the relevant variables from multivariable logistic regression analysis. By drawing a vertical line on each variable's scale, we can assign a score for each variable and calculate the total score for predicting the likelihood of developing an SSIs. By locating the corresponding position on the total score axis, we can estimate the probability of SSIs occurrence by connecting it to the probability axis.

## Discussion

SSIs are a significant concern in CRC patients undergoing surgery due to the high microbial load in the rectum and colon, as well as the potential for bacterial growth facilitated by the surgical procedure [3, 24]. The significant challenge posed by SSIs following colorectal cancer surgery has garnered attention worldwide. Between 2007 and 2010, the annual incidence of SSIs following colorectal cancer surgery was alarmingly high, reaching 20% [27]. However, surveys conducted under the auspices of the American College of Surgeons National Surgical Quality Improvement Program showed a decline in the incidence of colorectal SSIs from 17.58% in 2011 to 5.11% in 2015 [27, 28]. More recent data indicate an SSIs incidence ranging from 1.5% to 8.8% following colorectal surgery [4–6]. Our findings revealed an SSIs incidence of 7.65% following colorectal surgery, with SSIs significantly increasing treatment costs and hospital stays. Furthermore, independent risk factors for SSIs included having diabetes, undergoing an open surgical approach, and having a colostomy/ileostomy. These independent risk factors for SSIs are consistent with existing literature highlighting the influence of these factors on SSIs development [16–18, 29–33]. However, in our results, we did not find that high BMI and long surgical duration, although correlated with the occurrence of SSIs, were not independent risk factors for SSIs, which is consistent with another study conducted in China [34]. It is worth noting that EC Wick et al. also reported that obesity increases the incidence of SSIs, so the impact of weight on SSIs is worth further exploration [35].

We innovatively constructed a Nomogram plot, which is a visual tool used to estimate the individual risk of SSIs in patients.

Our study findings demonstrate that having a colostomy/ileostomy increases the incidence of postoperative SSIs, aligning with previous studies by Morikane et al. and Tang et al. [32, 33]. In clinical practice, it is worth exploring strategies to mitigate the risk of surgical site infections following ileostomy or colostomy reversal. Several randomized controlled trials have substantiated the efficacy of routine employment of negative pressure wound therapy in reducing the incidence of SSIs after ileostomy or colostomy reversal [36, 37]. The widespread adoption and practicality of this technique warrant further investigation.

Laparoscopic surgery is gradually becoming a common practice worldwide. Some studies have found that the laparoscopic approach significantly reduces the incidence of SSIs following colorectal surgery [38]. We observed an SSIs incidence of 6.14% in patients undergoing laparoscopic surgery, which is significantly lower than that in open surgery (13.5%). The role of laparoscopic techniques in reducing SSIs is commendable and warrants further promotion.

The recommendation for bowel preparation in CRC surgery is highly debated. Different studies and different countries provide varying conclusions and diverse recommendations.A recent meta-analysis of 38 RCTs, encompassing a total of 8458 patients, showed no significant difference in overall SSIs between MBP and no preparation [39]. This aligns with the findings of Katia F Güenaga et al., suggesting that bowel preparation can be safely omitted in colon surgery without a significant reduction in complication rates [39, 40]. Furthermore, the report indicates that compared to MBP only or no preparation, MBP with OABP significantly reduces the overall occurrence of SSIs [39]. However, the contrast with OABP only does not yield a significant effect [39]. The controversy arises in whether to combine OABP with MBP. A recent meta-analysis by Chen et al. compared the effectiveness of MBP alone, OABP alone, and the combination of MBP and OABP, indicating a reduction in infectious complications with the combination [41]. Three randomized controlled trials exploring the efficacy comparison between MBP and OABP alone versus OABP alone did not show statistically significant differences [42–44]. Although recent studies by Ravi Pokala Kiran et al. and Aaron L Klinger et al. suggested that the combination of MBP and OABP is associated with the lowest risk of infectious complications [45, 46]. Both the American Society for Enhanced Recovery and the Perioperative Quality Initiative joint consensus and the 2022 Chinese guidelines for SSIs recommend preoperative oral antibiotics combined with MBP to reduce the risk of SSIs in adult patients undergoing elective colorectal surgery [47, 48]. They do not recommend performing mechanical bowel preparation alone (without oral antibiotics) in adult patients undergoing elective colorectal surgery. The Australian guidelines and European ERAS guidelines suggest that routine use of MBP should not be employed in colon surgery [49, 50]. Therefore, whether bowel preparation is recommended in CRC surgery, and if so, the recommended method of bowel preparation, may not have a uniform answer in different regions. It is necessary for different regions to conduct high-quality RCT research. Our study results did not show significant statistical differences in the incidence of SSIs among these methods, which needs to be further explored in subsequent multicenter RCT studies.

While we observed an increase in SSIs rates during the COVID-19 pandemic, it did not reach statistical significance. This phenomenon may be attributed to the sudden emergence of the COVID-19 virus, which placed a significant burden on healthcare institutions and posed substantial challenges to infection prevention efforts. It is worth noting that all patients undergoing surgery during the pandemic period in our study had tested negative for COVID-19 within the month prior to surgery. A study conducted in Germany indicated a reduction in surgical site infection rates, attributing it to strict hygiene measures during the COVID-19 period, including continuous use of masks and gloves, reinforced hygiene standards for clinical examinations, and routine wound control [19].

Despite the significant findings, this study has some limitations. Firstly, its retrospective design introduces inherent limitations, such as selection bias and potential incomplete data. In terms of patient-related factors, we did not delve into biochemical indicators such as hemoglobin, lbumin, etc. Concerning surgical factors, we did not incorporate the ASA score. As for environmental factors, our procedures adhere to standardized protocols. These factors' impact need to be considered in subsequent studies. Secondly, the study was conducted at a single institution, which may limit the generalizability of the results. Multi-center studies involving diverse patient populations would provide more robust evidence. Additionally, the influence of other unmeasured confounding factors on SSIs occurrence could not be completely excluded, such as American Society of Anesthesiology score, nutritional status, immune system status. Future studies may benefit from more comprehensive data collection to further address these potential confounders.

## Conclusions

This study showed that the incidence of SSIs after colorectal surgery was 7.65%. the presence of diabetes, open surgical procedures, and the presence of colostomy/ileostomy were probably associated with the occurrence of SSIs after colorectal surgery. A nomogram plot was developed based on this to facilitate clinical application. Considering the limitations of this observational study, multicenter randomized controlled trials are still needed to further determine the risk factors for SSIs after colorectal surgery.

## Data Availability

The datasets generated in the current study are not publicly available. However, the datasets used or analyzed in the study will be made available by the corresponding author upon reasonable request.

## Abbreviations

CRC: Colorectal cancer
SSIs: Surgical site infections
BMI: Body mass index
Cis: Confidence intervals
ORs: Odds ratios
ORAB: Oral antibiotics preparation
MBP: Mechanical bowel preparation only
ORAB + MBP: Combined mechanical and oral antibiotics

## References

[1] Morgan E, Arnold M, Gini A, Lorenzoni V, Cabasag C, Laversanne M, Vignat J, Ferlay J, Murphy N, Bray F (2023). Global burden of colorectal cancer in 2020 and 2040: Incidence and mortality estimates from GLOBOCAN. Gut.

[2] Siegel RL, Miller KD, Wagle NS, Jemal A (2023). Cancer statistics, 2023. Ca Cancer J Clin.

[3] Vo E, Massarweh NN, Chai CY, Cao HST, Zamani N, Abraham S, Adigun K, Awad SS (2018). Association of the addition of oral antibiotics to mechanical bowel preparation for left colon and rectal cancer resections with reduction of surgical site infections. JAMA Surg.

[4] Zhang X, Yang Y, Liu P, Wang P, Li X, Zhu J, et al. Identification of risk factors and phenotypes of surgical site infection in patients after abdominal surgery. Ann Surg. 2023;278(5):e988–94. 10.1097/SLA.0000000000005939 37309899

[5] Nasser H, Ivanics T, Leonard-Murali S, Stefanou A (2020). Risk factors for surgical site infection after laparoscopic colectomy: An NSQIP database analysis. J Surg Res.

[6] Levy BE, Wilt WS, Castle JT, McAtee E, Walling SC, Davenport DL, Bhakta A, Patel JA (2023). Surgical site infections in colorectal resections: what is the cost?. J Surg Res.

[7] Kamboj M, Childers T, Sugalski J, Antonelli D, Bingener-Casey J, Cannon J, Cluff K, Davis KA, Dellinger EP, Dowdy SC: Risk of surgical site infection (SSI) following colorectal resection is higher in patients with disseminated cancer: an NCCN member cohort study. Infect Control Hosp Epidemiol 2018, 39(5):555–562. 10.1017/ice.2018.40 PMC6707075 29553001

[8] Nobuhara H, Matsugu Y, Soutome S, Hayashida S, Hasegawa T, Akashi M, Yamada S-i, Kurita H, Nakahara H, Nakahara M (2022). Perioperative oral care can prevent surgical site infection after colorectal cancer surgery: A multicenter, retrospective study of 1,926 cases analyzed by propensity score matching. Surgery.

[9] Smith RL, Bohl JK, McElearney ST, Friel CM, Barclay MM, Sawyer RG, Foley EF (2004). Wound infection after elective colorectal resection. Ann Surg.

[10] Cima R, Dankbar E, Lovely J, Pendlimari R, Aronhalt K, Nehring S, Hyke R, Tyndale D, Rogers J, Quast L (2013). Colorectal surgery surgical site infection reduction program: a national surgical quality improvement program–driven multidisciplinary single-institution experience. J Am Coll Surg.

[11] Badia J, Casey A, Petrosillo N, Hudson P, Mitchell S, Crosby C (2017). Impact of surgical site infection on healthcare costs and patient outcomes: a systematic review in six European countries. J Hosp Infect.

[12] Tartari E, Weterings V, Gastmeier P, Rodríguez Baño J, Widmer A, Kluytmans J, Voss A (2017). Patient engagement with surgical site infection prevention: an expert panel perspective. Antimicrob Resist Infect Control.

[13] Saleh K, Schmidtchen A (2015). Surgical site infections in dermatologic surgery: etiology, pathogenesis, and current preventative measures. Dermatol Surg.

[14] Owens C, Stoessel K (2008). Surgical site infections: epidemiology, microbiology and prevention. J Hosp Infect.

[15] Allegranzi B, Bischoff P, de Jonge S, Kubilay NZ, Zayed B, Gomes SM, Abbas M, Atema JJ, Gans S, van Rijen M (2016). New WHO recommendations on preoperative measures for surgical site infection prevention: an evidence-based global perspective. Lancet Infect Dis.

[16] Cheng CW, Cizik AM, Dagal AH, Lewis L, Lynch J, Bellabarba C, Bransford RJ, Zhou H (2019). Body mass index and the risk of deep surgical site infection following posterior cervical instrumented fusion. Spine J.

[17] Meijs AP, Koek MB, Vos MC, Geerlings SE, Vogely HC, de Greeff SC (2019). The effect of body mass index on the risk of surgical site infection. Infect Control Hosp Epidemiol.

[18] Martin ET, Kaye KS, Knott C, Nguyen H, Santarossa M, Evans R, Bertran E, Jaber L (2016). Diabetes and risk of surgical site infection: a systematic review and meta-analysis. Infect Control Hosp Epidemiol.

[19] Chacón-Quesada T, Rohde V, von der Brelie C (2021). Less surgical site infections in neurosurgery during COVID-19 times-one potential benefit of the pandemic?. Neurosurg Rev.

[20] Chen M, Song X (2016). Chen L-z, Lin Z-d, Zhang X-l: Comparing mechanical bowel preparation with both oral and systemic antibiotics versus mechanical bowel preparation and systemic antibiotics alone for the prevention of surgical site infection after elective colorectal surgery. Dis Colon Rectum.

[21] Yao L, Xiao M, Luo Y, Yan L, Zhao Q, Li Y (2021). Research on the factors that influence patients with colorectal cancer participating in the prevention and control of surgical site infection: Based on the extended theory of planned behaviour. Health Expect.

[22] Tang Y, Zhang R, Yang W, Li W, Tao K (2020). Prognostic value of surgical site infection in patients after radical colorectal cancer resection. Med Sci Mon.

[23] Weiser MR (2018). AJCC 8th Edition: Colorectal Cancer. Ann Surg Oncol.

[24] Anderson DJ (2011). Surgical site infections. Infect Dis Clin.

[25] Park SY (2018). Nomogram: an analogue tool to deliver digital knowledge. J Thorac Cardiovasc Surg.

[26] Grömping U (2015). Using R and RStudio for data management, statistical analysis and graphics. J Stat Softw.

[27] Pujol M, Limón E, López-Contreras J, Sallés M, Bella F, Gudiol F (2012). Surveillance of surgical site infections in elective colorectal surgery. Results of the VINCat Program (2007–2010). Enferm Infecc Microbiol Clin.

[28] DeHaas D, Aufderheide S, Gano J, Weigandt J, Ries J, Faust B (2016). Colorectal surgical site infection reduction strategies. Am J Surg.

[29] Gibbons C, Bruce J, Carpenter J, Wilson AP, Wilson J, Pearson A, et al. Identification of risk factors by systematic review and development of risk-adjusted models for surgical site infection. Health Technol Assess. 2011;15(30):1–156, iii–iv. 10.3310/hta15300 21884656

[30] Imai E, Ueda M, Kanao K, Kubota T, Hasegawa H, Omae K, Kitajima M (2008). Surgical site infection risk factors identified by multivariate analysis for patient undergoing laparoscopic, open colon, and gastric surgery. Am J Infect Control.

[31] Leong G, Wilson J, Charlett A (2006). Duration of operation as a risk factor for surgical site infection: comparison of English and US data. J Hosp Infect.

[32] Tang R, Chen HH, Wang YL, Changchien CR, Chen JS, Hsu KC, Chiang JM, Wang JY (2001). Risk factors for surgical site infection after elective resection of the colon and rectum: a single-center prospective study of 2,809 consecutive patients. Ann Surg.

[33] Morikane K, Honda H, Yamagishi T, Suzuki S, Aminaka M (2014). Factors associated with surgical site infection in colorectal surgery: the Japan nosocomial infections surveillance. Infect Control Hosp Epidemiol.

[34] Zhang X, Wang Z, Chen J, Wang P, Luo S, Xu X, Mai W, Li G, Wang G, Wu X (2020). Incidence and risk factors of surgical site infection following colorectal surgery in China: a national cross-sectional study. BMC Infect Dis.

[35] Wick EC, Hirose K, Shore AD, Clark JM, Gearhart SL, Efron J, Makary MA (2011). Surgical site infections and cost in obese patients undergoing colorectal surgery. Arch Surg.

[36] Low EZ, Nugent TS, O'Sullivan NJ, Kavanagh D, Larkin JO, McCormick PH, Mehigan BJ, Kelly ME (2022). Application of PREVENA (Surgical Incision Protection System) in reducing surgical site infections following reversal of ileostomy or colostomy: the PRIC study protocol. Int J Colorectal Dis.

[37] Carrano FM, Maroli A, Carvello M, Foppa C, Sacchi M, Crippa J, et al. Negative-pressure wound therapy after stoma reversal in colorectal surgery: a randomized controlled trial. BJS Open. 2021;5(6):zrab116. 10.1093/bjsopen/zrab116 PMC8669787 34904647

[38] Kiran RP, El-Gazzaz GH, Vogel JD, Remzi FH (2010). Laparoscopic approach significantly reduces surgical site infections after colorectal surgery: data from national surgical quality improvement program. J Am Coll Surg.

[39] Toh JWT, Phan K, Hitos K, Pathma-Nathan N, El-Khoury T, Richardson AJ, Morgan G, Engel A, Ctercteko G (2018). Association of mechanical bowel preparation and oral antibiotics before elective colorectal surgery with surgical site infection: a network meta-analysis. JAMA Netw Open.

[40] Güenaga KF, Matos D, Wille-Jørgensen P: Mechanical bowel preparation for elective colorectal surgery. Cochrane Database Syst Rev 2011;2011(9):Cd001544. 10.1002/14651858.CD001544.pub4 PMC7066937 21901677

[41] Chen M, Song X, Chen LZ, Lin ZD, Zhang XL (2016). Comparing mechanical bowel preparation with both oral and systemic antibiotics versus mechanical bowel preparation and systemic antibiotics alone for the prevention of surgical site infection after elective colorectal surgery: a meta-analysis of randomized controlled clinical trials. Dis Colon Rectum.

[42] Zmora O, Mahajna A, Bar-Zakai B, Rosin D, Hershko D, Shabtai M, Krausz MM, Ayalon A (2003). Colon and rectal surgery without mechanical bowel preparation: a randomized prospective trial. Ann Surg.

[43] Zmora O, Mahajna A, Bar-Zakai B, Hershko D, Shabtai M, Krausz MM, Ayalon A (2006). Is mechanical bowel preparation mandatory for left-sided colonic anastomosis? Results of a prospective randomized trial. Tech Coloproctol.

[44] Reddy BS, Macfie J, Gatt M, Larsen CN, Jensen SS, Leser TD (2007). Randomized clinical trial of effect of synbiotics, neomycin and mechanical bowel preparation on intestinal barrier function in patients undergoing colectomy. Br J Surg.

[45] Kiran RP, Murray AC, Chiuzan C, Estrada D, Forde K: Combined preoperative mechanical bowel preparation with oral antibiotics significantly reduces surgical site infection, anastomotic leak, and ileus after colorectal surgery. Ann Surg 2015;262(3):416–425; discussion 423–415. 10.1097/SLA.0000000000001416 26258310

[46] Klinger AL, Green H, Monlezun DJ, Beck D, Kann B, Vargas HD, Whitlow C, Margolin D (2019). The role of bowel preparation in colorectal surgery: Results of the 2012–2015 ACS-NSQIP Data. Ann Surg.

[47] Chinese guideline for the prevention of surgical site infection. Zhonghua Wei Chang Wai Ke Za Zhi. 2019;22(4):301–14. 10.3760/cma.j.issn.1671-0274.2019.04.001 31054543

[48] Holubar SD, Hedrick T, Gupta R, Kellum J, Hamilton M, Gan TJ, Mythen MG, Shaw AD, Miller TE (2017). American Society for Enhanced Recovery (ASER) and Perioperative Quality Initiative (POQI) joint consensus statement on prevention of postoperative infection within an enhanced recovery pathway for elective colorectal surgery. Perioper Med (Lond).

[49] James SL, Castle CD, Dingels ZV, Fox JT, Hamilton EB, Liu Z, Roberts NLS, Sylte DO, Bertolacci GJ, Cunningham M (2020). Estimating global injuries morbidity and mortality: methods and data used in the Global Burden of Disease 2017 study. Inj Prev.

[50] Gustafsson UO, Scott MJ, Schwenk W, Demartines N, Roulin D, Francis N, McNaught CE, MacFie J, Liberman AS, Soop M (2012). Guidelines for perioperative care in elective colonic surgery: Enhanced Recovery After Surgery (ERAS®) Society recommendations. Clin Nutr.

